# *catena*-Poly[[silver(I)-μ-4-aminopyridine] perchlorate]: a 1-D staircase coordination polymer

**DOI:** 10.1107/S1600536810037682

**Published:** 2010-09-30

**Authors:** Ryan K. Golder, Christopher M. Fitchett, Jan L. Wikaira, Peter J. Steel

**Affiliations:** a Chemistry Department, University of Canterbury, PO Box 4800, Christchurch, New Zealand

## Abstract

Reaction of 4-amino­pyridine with silver(I) perchlorate leads to a one-dimensional coordination polymer, {[Ag(C_5_H_6_N_2_)]ClO_4_}_*n*_, in which the amino­pyridine binds through both N atoms. The perchlorate anion is hydrogen bonded to the amino H atoms and inter­acts weakly with the silver(I) atoms (Ag—O > 2.70 Å), both located on inversion centres, and some aromatic H atoms (O—H > 2.55 ÅA), thereby extending the dimensionality of the assembly. This is the first silver complex in which this ligand acts in a bridging mode.

## Related literature

For discrete silver complexes of the same ligand, see: Kristian­sson (2000 ▶); Abu-Youssef *et al.* (2006 ▶); Liu *et al.* (2005 ▶); Zhu *et al.* (2003*a* ▶,*b* ▶); Li *et al.* (2005 ▶); Ma *et al.* (2004 ▶). For metallosupra­molecular assemblies derived from bridging heterocyclic ligands, see: Steel (2005 ▶). For the use of silver(I) for the self-assembly of both discrete and polymeric aggregates with diverse mol­ecular architectures, see: Fitchett & Steel (2006 ▶); O’Keefe & Steel (2007 ▶). For a review of the use of pyrazine and analogues as bridging ligands for silver(I)-based assemblies, see: Steel & Fitchett (2008 ▶).
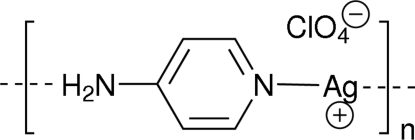


## Experimental

### 

#### Crystal data

[Ag(C_5_H_6_N_2_)]ClO_4_
*M* *_r_* = 301.44
Triclinic, 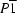

*a* = 5.0720 (2) Å
*b* = 9.0025 (3) Å
*c* = 9.5520 (3) Å
α = 93.198 (2)°
β = 96.992 (2)°
γ = 100.452 (2)°
*V* = 424.37 (3) Å^3^
*Z* = 2
Mo *K*α radiation
μ = 2.67 mm^−1^
*T* = 113 K
0.35 × 0.11 × 0.05 mm

#### Data collection

Bruker APEXII CCD diffractometer
Absorption correction: multi-scan (*SADABS*; Bruker, 2009 ▶) *T*_min_ = 0.455, *T*_max_ = 0.878
9107 measured reflections
1740 independent reflections
1591 reflections with *I* > 2σ(*I*)
*R*_int_ = 0.046

#### Refinement

*R*[*F* ^2^ > 2σ(*F* ^2^)] = 0.021
*wR*(*F* ^2^) = 0.051
*S* = 1.03
1740 reflections
127 parameters
H atoms treated by a mixture of independent and constrained refinement
Δρ_max_ = 0.57 e Å^−3^
Δρ_min_ = −0.78 e Å^−3^

### 

Data collection: *APEX2* (Bruker, 2009 ▶); cell refinement: *SAINT* (Bruker, 2009 ▶); data reduction: *SAINT*; program(s) used to solve structure: *SHELXS97* (Sheldrick, 2008 ▶); program(s) used to refine structure: *SHELXL97* (Sheldrick, 2008 ▶); molecular graphics: *SHELXTL* (Sheldrick, 2008 ▶); software used to prepare material for publication: *SHELXTL*.

## Supplementary Material

Crystal structure: contains datablocks global, I. DOI: 10.1107/S1600536810037682/bv2161sup1.cif

Structure factors: contains datablocks I. DOI: 10.1107/S1600536810037682/bv2161Isup2.hkl

Additional supplementary materials:  crystallographic information; 3D view; checkCIF report

## Figures and Tables

**Table 1:** Hydrogen-bond geometry (Å, °)

*D*—H⋯*A*	*D*—H	H⋯*A*	*D*⋯*A*	*D*—H⋯*A*
N2—H2*C*⋯O1^i^	0.86 (3)	2.16 (3)	2.984 (3)	161 (2)
N2—H2*B*⋯O3^ii^	0.85 (3)	2.29 (3)	2.984 (3)	139 (2)

Symmetry codes: (i) 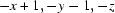; (ii) 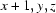.

**Fig. 1.**
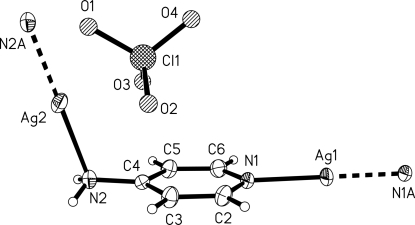
The molecular structure of (2), showing displacement ellipsoids at the 50% probability level.

**Fig. 2.**
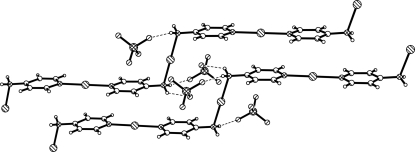
Extended structure of (2), showing the staircase structure and the hydrogen bonding interactions.

## supplementary crystallographic information

### Comment

For some time we have been involved in the study of metallosupramolecular assemblies derived from bridging heterocyclic ligands (Steel, 2005). In recent years we have focused on the use of silver(I) for the self-assembly of both discrete and polymeric aggregates with diverse molecular architectures (Fitchett & Steel, 2006; O'Keefe & Steel, 2007). In this context, we have recently reviewed the use of pyrazine and analogues as bridging ligands for silver(I)-based assemblies (Steel & Fitchett, 2008). 4-Aminopyridine (1) is a less symmetrical ligand that can potentially act as a bridge between metal centres. X-ray structures have been reported for complexes of (1) with silver nitrate (Kristiansson, 2000; Abu-Youssef *et al.*, 2006), silver bicarbonate (Liu *et al.*, 2005), silver trifluoroacetate (Zhu *et al.*, 2003*a*), silver trifluoromethanesulfonate (Zhu *et al.*, 2003*b*; Liu *et al.*, 2005), silver terephthalate (Li *et al.*, 2005) and silver 3-nitrobenzoate (Ma *et al.*, 2004). However, in all these cases the ligand acts as a monodentate ligand binding through the pyridine nitrogen only and therefore forms discrete coordination complexes. We now describe a one-dimensional coordination polymer, obtained from reaction between this ligand and silver perchlorate, in which ligand (1) acts in a bridging bidentate mode.

The complex (2) crystallizes in the triclinic space group P-1 with a full 4-aminopyridine ligand, two half silver atoms and a perchlorate anion in the asymmetric unit (Fig. 1). The two independent silver atoms each lie on crystallographic centres of inversion and therefore act as linear connectors resulting in a 1-D coordination polymer. Ligand (1) coordinates to Ag1 through two pyridine N atoms and to Ag2 *via* two amino N atoms. The resulting coordination polymer has a staircase-type structure that results from the fact that the amino nitrogen introduces an angular turn (C4—N2—Ag2 111.9 (1)°) into the polymer chain.

Both of the amino group H atoms are hydrogen bonded (Table 1) to adjacent perchlorate counterions, which in turn serve to bridge adjacent chains through two such hydrogen bonds (Fig. 2). The perchlorate O atoms are also involved in weak interactions with the silver atoms, which in the case of Ag2 leads to a pseudo-octahedral coordination environment for this atom. The perchlorate O atoms make weak contacts with some CH H atoms. These additional interactions increase the dimensionality of the overall assembly. This structure represents the first example in which ligand (1) acts as a bridging ligand for silver(I).

### Experimental

The title compound was prepared by slow evaporation of an acetone solution containing an equimolar ratio of 4-aminopyridine and silver perchlorate.

### Refinement

CH hydrogen atoms were introduced in calculated positions as riding atoms, with *U*_iso_(H) = 1.2*U*_eq_(C). The NH H atoms were located from a difference Fourier map and their positions refined with *U*_iso_(H) = 1.2*U*_eq_(N).

### Crystal data

**Table:**

[Ag(C_5_H_6_N_2_)]ClO_4_	*Z* = 2
*M**_r_* = 301.44	*F*(000) = 292
Triclinic, *P*1	*D*_x_ = 2.359 Mg m^−^^3^
Hall symbol: -P 1	Mo *K*α radiation, λ = 0.71073 Å
*a* = 5.0720 (2) Å	Cell parameters from 6232 reflections
*b* = 9.0025 (3) Å	θ = 3.0–26.4°
*c* = 9.5520 (3) Å	µ = 2.67 mm^−^^1^
α = 93.198 (2)°	*T* = 113 K
β = 96.992 (2)°	Prism, orange
γ = 100.452 (2)°	0.35 × 0.11 × 0.05 mm
*V* = 424.37 (3) Å^3^	

### Data collection

**Table:**

Bruker APEXII CCD diffractometer	1740 independent reflections
Radiation source: fine-focus sealed tube	1591 reflections with *I* > 2σ(*I*)
graphite	*R*_int_ = 0.046
φ and ω scans	θ_max_ = 26.4°, θ_min_ = 2.3°
Absorption correction: multi-scan (*SADABS*; Bruker, 2009)	*h* = −6→6
*T*_min_ = 0.455, *T*_max_ = 0.878	*k* = −11→11
9107 measured reflections	*l* = −11→11

### Refinement

**Table:**

Refinement on *F*^2^	Primary atom site location: structure-invariant direct methods
Least-squares matrix: full	Secondary atom site location: difference Fourier map
*R*[*F*^2^ > 2σ(*F*^2^)] = 0.021	Hydrogen site location: inferred from neighbouring sites
*wR*(*F*^2^) = 0.051	H atoms treated by a mixture of independent and constrained refinement
*S* = 1.03	*w* = 1/[σ^2^(*F*_o_^2^) + (0.0333*P*)^2^ + 0.1489*P*] where *P* = (*F*_o_^2^ + 2*F*_c_^2^)/3
1740 reflections	(Δ/σ)_max_ = 0.017
127 parameters	Δρ_max_ = 0.57 e Å^−^^3^
0 restraints	Δρ_min_ = −0.78 e Å^−^^3^

### Special details

**Table:**

Geometry. All e.s.d.'s (except the e.s.d. in the dihedral angle between two l.s. planes) are estimated using the full covariance matrix. The cell e.s.d.'s are taken into account individually in the estimation of e.s.d.'s in distances, angles and torsion angles; correlations between e.s.d.'s in cell parameters are only used when they are defined by crystal symmetry. An approximate (isotropic) treatment of cell e.s.d.'s is used for estimating e.s.d.'s involving l.s. planes.
Refinement. Refinement of *F*^2^ against ALL reflections. The weighted *R*-factor *wR* and goodness of fit *S* are based on *F*^2^, conventional *R*-factors *R* are based on *F*, with *F* set to zero for negative *F*^2^. The threshold expression of *F*^2^ > σ(*F*^2^) is used only for calculating *R*-factors(gt) *etc*. and is not relevant to the choice of reflections for refinement. *R*-factors based on *F*^2^ are statistically about twice as large as those based on *F*, and *R*- factors based on ALL data will be even larger.

### Fractional atomic coordinates and isotropic or equivalent isotropic displacement parameters (Å^2^)

**Table:**

	*x*	*y*	*z*	*U*_iso_*/*U*_eq_	
Ag1	0.0000	0.0000	0.5000	0.02748 (9)	
Ag2	0.5000	−0.5000	0.0000	0.02716 (9)	
Cl1	−0.04375 (9)	−0.70217 (6)	0.19452 (5)	0.02471 (12)	
O1	−0.0156 (4)	−0.81713 (18)	0.08849 (17)	0.0372 (4)	
O2	0.0718 (4)	−0.7384 (2)	0.32954 (17)	0.0405 (4)	
O3	0.0969 (3)	−0.55690 (18)	0.16137 (19)	0.0338 (4)	
O4	−0.3233 (3)	−0.6953 (2)	0.1977 (2)	0.0530 (6)	
N1	0.2684 (3)	−0.10407 (19)	0.38878 (18)	0.0212 (3)	
C2	0.3565 (4)	−0.2298 (2)	0.4306 (2)	0.0253 (4)	
H2A	0.3003	−0.2710	0.5139	0.030*	
C3	0.5233 (4)	−0.3006 (2)	0.3584 (2)	0.0245 (4)	
H3A	0.5804	−0.3886	0.3917	0.029*	
C4	0.6080 (4)	−0.2422 (2)	0.2358 (2)	0.0187 (4)	
C5	0.5244 (4)	−0.1101 (2)	0.1946 (2)	0.0224 (4)	
H5A	0.5824	−0.0647	0.1134	0.027*	
C6	0.3576 (4)	−0.0467 (2)	0.2725 (2)	0.0246 (4)	
H6A	0.3017	0.0430	0.2427	0.030*	
N2	0.7619 (3)	−0.3164 (2)	0.15325 (19)	0.0218 (3)	
H2B	0.874 (5)	−0.362 (3)	0.199 (3)	0.026*	
H2C	0.845 (5)	−0.259 (3)	0.097 (3)	0.026*	

### Atomic displacement parameters (Å^2^)

**Table:**

	*U*^11^	*U*^22^	*U*^33^	*U*^12^	*U*^13^	*U*^23^
Ag1	0.01995 (12)	0.03359 (15)	0.02912 (14)	0.00871 (9)	0.00516 (9)	−0.01157 (10)
Ag2	0.02874 (13)	0.02542 (14)	0.02684 (14)	0.00551 (9)	0.00580 (9)	−0.00782 (9)
Cl1	0.0227 (2)	0.0299 (3)	0.0266 (3)	0.01105 (19)	0.00968 (18)	0.0130 (2)
O1	0.0491 (10)	0.0312 (9)	0.0296 (9)	0.0013 (7)	0.0083 (7)	0.0022 (7)
O2	0.0520 (10)	0.0550 (11)	0.0238 (8)	0.0281 (9)	0.0100 (7)	0.0138 (8)
O3	0.0347 (8)	0.0259 (8)	0.0473 (10)	0.0105 (6)	0.0211 (7)	0.0102 (7)
O4	0.0238 (9)	0.0665 (13)	0.0815 (15)	0.0196 (8)	0.0229 (9)	0.0445 (12)
N1	0.0193 (8)	0.0232 (8)	0.0211 (8)	0.0061 (6)	0.0032 (6)	−0.0055 (7)
C2	0.0309 (11)	0.0270 (11)	0.0197 (10)	0.0059 (8)	0.0089 (8)	0.0023 (8)
C3	0.0322 (11)	0.0224 (10)	0.0225 (10)	0.0112 (8)	0.0075 (8)	0.0045 (8)
C4	0.0167 (9)	0.0202 (9)	0.0183 (9)	0.0031 (7)	0.0015 (7)	−0.0025 (7)
C5	0.0276 (10)	0.0207 (10)	0.0204 (10)	0.0058 (8)	0.0063 (8)	0.0037 (8)
C6	0.0270 (10)	0.0209 (10)	0.0269 (11)	0.0088 (8)	0.0020 (8)	0.0000 (8)
N2	0.0212 (8)	0.0233 (9)	0.0227 (9)	0.0069 (7)	0.0072 (7)	0.0004 (7)

### Geometric parameters (Å, °)

**Table:**

Ag1—N1	2.1363 (16)	C2—H2A	0.9500
Ag1—N1^i^	2.1363 (16)	C3—C4	1.393 (3)
Ag2—N2^ii^	2.2582 (18)	C3—H3A	0.9500
Ag2—N2	2.2583 (18)	C4—C5	1.394 (3)
Cl1—O2	1.4301 (16)	C4—N2	1.399 (3)
Cl1—O4	1.4344 (16)	C5—C6	1.371 (3)
Cl1—O3	1.4430 (16)	C5—H5A	0.9500
Cl1—O1	1.4454 (17)	C6—H6A	0.9500
N1—C6	1.344 (3)	N2—H2B	0.85 (3)
N1—C2	1.353 (3)	N2—H2C	0.86 (3)
C2—C3	1.373 (3)		
			
N1—Ag1—N1^i^	180.00 (5)	C4—C3—H3A	120.3
N2^ii^—Ag2—N2	180.0	C5—C4—C3	117.63 (18)
O2—Cl1—O4	109.54 (11)	C5—C4—N2	120.84 (18)
O2—Cl1—O3	109.91 (12)	C3—C4—N2	121.47 (18)
O4—Cl1—O3	108.88 (10)	C6—C5—C4	119.20 (19)
O2—Cl1—O1	108.65 (11)	C6—C5—H5A	120.4
O4—Cl1—O1	110.77 (13)	C4—C5—H5A	120.4
O3—Cl1—O1	109.08 (10)	N1—C6—C5	123.77 (19)
C6—N1—C2	116.76 (17)	N1—C6—H6A	118.1
C6—N1—Ag1	120.93 (13)	C5—C6—H6A	118.1
C2—N1—Ag1	122.31 (13)	C4—N2—Ag2	111.91 (12)
N1—C2—C3	123.15 (19)	C4—N2—H2B	115.5 (17)
N1—C2—H2A	118.4	Ag2—N2—H2B	104.1 (17)
C3—C2—H2A	118.4	C4—N2—H2C	113.7 (17)
C2—C3—C4	119.45 (19)	Ag2—N2—H2C	101.4 (17)
C2—C3—H3A	120.3	H2B—N2—H2C	109 (2)

Symmetry codes: (i) −*x*, −*y*, −*z*+1; (ii) −*x*+1, −*y*−1, −*z*.

### Hydrogen-bond geometry (Å, °)

**Table:**

*D*—H···*A*	*D*—H	H···*A*	*D*···*A*	*D*—H···*A*
N2—H2C···O1^ii^	0.86 (3)	2.16 (3)	2.984 (3)	161 (2)
N2—H2B···O3^iii^	0.85 (3)	2.29 (3)	2.984 (3)	139 (2)

Symmetry codes: (ii) −*x*+1, −*y*−1, −*z*; (iii) *x*+1, *y*, *z*.
